# Effects of Dietary Ferric EDTA Levels on Vegetables and Mirror Carp (*Cyprinus carpio var*. *specularis*) in Aquaponics System

**DOI:** 10.3390/ani15060792

**Published:** 2025-03-11

**Authors:** Yu Liu, Zhipeng Dou, Chengwei Ji, Qingbo Zhou, Jun Zhao, Ke Wang, Chao Chen, Qing Liu

**Affiliations:** 1 College of Animal Science, Shanxi Agricultural University, Jinzhong 030800, China; 18342255015@163.com (Y.L.); 17090856662@163.com (Z.D.); 18569627325@163.com (C.J.); lucas.z1997@icloud.com (Q.Z.); zj13613414986@163.com (J.Z.); 15364896278@163.com (K.W.); 2 Shanxi Key Laboratory of Animal Genetics Resource Utilization and Breeding, Jinzhong 030800, China; 3 College of Materials Science and Engineering, Taiyuan University of Technology, Taiyuan 030024, China; chenchao@tyut.edu.cn

**Keywords:** aquaponics system, ferric EDTA, mirror carp (*Cyprinus carpio var. specularis*)

## Abstract

This study investigated the effects of dietary iron supplementation on water quality, plant growth, and fish health in an aquaponic system over 90 days. Iron supplementation significantly improved plant growth, with increased plant height, stem diameter, leaf count, and fruit yield in tomatoes (*Solanum lycopersicum*) and pak choi (*Brassica rapa subsp. Chinensis*) (*p* < 0.05). The water pH fluctuated with varying iron content, and higher iron levels promoted better plant growth by improving iron availability (*p* < 0.05). During the first 60 days, all red blood cell counts and hemoglobin levels increased, but the growth and nutritional composition of mirror carp (*Cyprinus carpio var. specularis*) showed no significant differences. By day 90, fish in the T3 group (800 mg/kg iron) exhibited significantly reduced growth and feed conversion rates (*p* < 0.05). Histological analysis of liver tissue indicated iron-induced liver damage; additionally, excessive iron intake suppressed erythropoiesis, leading to lower red blood cell counts and hemoglobin levels (*p* < 0.05). The results indicate that moderate iron supplementation improves plant growth, but excessive iron can negatively impact fish health, particularly liver function and blood formation. These findings provide valuable insights for optimizing iron levels in aquaponic systems.

## Simple Summary

This study investigated the effects of dietary iron supplementation on water quality, plant growth, and fish health in an aquaponic system over 90 days. Iron supplementation significantly improved plant growth, with increased plant height, stem diameter, leaf count, and fruit yield in tomatoes (*Solanum lycopersicum*) and pak choi (*Brassica rapa subsp. chinensis*) (*p* < 0.05). Moderate iron supplementation (200 mg/kg and 400 mg/kg) boosted fish health, including better blood cell production and immunity. However, too much iron (800 mg/kg) caused liver damage and stunted fish growth. The results suggest that feeding 200 mg/kg of iron for 60 days is optimal for both plant and fish growth in a sustainable aquaponic system.

## 1. Introduction

In aquaponic systems, plants primarily obtain nutrients from fish waste [1]. Aquaculture effluents are rich in nitrogen and phosphorus, derived from fish gill excretion, feces, and leftover feed [2,3]. However, despite the presence of these nutrients, certain mineral elements, particularly iron (Fe), are deficient in aquaponic systems, leading to plant growth deficiencies [4,5].

Iron deficiency in fish slows their growth and increases their susceptibility to anemia. Previous studies have demonstrated the critical role of iron in fish health. For instance, iron deficiency affects the lipid metabolism in yellow catfish *(Pelteobagrus fulvidraco)* liver and reduces the activity of various antioxidant enzymes in the liver [6]. Iron is also vital for immune activity in fish. Iron deficiency can impair phagocyte function; insufficient dietary iron increases the susceptibility of channel catfish(*Ictalurus punctatus*) to bacterial infections [7], whereas iron-supplemented diets significantly enhance macrophage migration in this species [8]. Serum superoxide dismutase (SOD) activity correlates with serum iron levels [9], and lysozyme activity serves as a reference for fish immunity [10]. Iron-enriched feeds have been commercially adopted, with studies indicating that 580–1200 mg Fe/kg feed optimally promotes growth and improves hematological parameters in Nile tilapia (*Oreochromis niloticus*) [11]. However, exceeding 1500 mg/kg iron in feed negatively affects the growth and immunity of channel catfish (*Ictalurus punctatus*) [12].

The bioavailability of iron depends on its chemical form, such as Fe^3+^ and Fe^2+^, which can be from inorganic or organic complexes [13]. Hence, the form of iron in feed profoundly affects its bioavailability. While fish can absorb iron through their gills, most of the iron absorption occurs in the gut [13]. Excess iron can be transferred via transferrin to the gills and subsequently released into the water, where it becomes available for plant use in aquaponic systems [14]. In bony fish, the gut serves as the primary absorption site for Fe, zinc, and copper, and intestinal regulation maintains the homeostasis of these metals [15]. Craig et al. also highlighted the competitive interactions between iron and copper absorption in zebrafish (*Danio rerio*) [16].

In fish, serum transferrin serves as the primary transporter of iron, moving iron ions from storage sites to erythrocytes for hemoglobin synthesis or other iron-requiring tissues [17]. The body lacks an active iron excretion mechanism and maintains the iron balance by regulating intestinal absorption, recycling iron from senescent erythrocytes, and releasing iron from mononuclear macrophages [18]. Hepcidin, a hormone regulating iron homeostasis, senses and responds to changes in blood iron levels. Transferrin and hepcidin also play roles in fish immunity; for example, hepcidin has been identified as an antimicrobial peptide [19], and transferrin expression increases during parasitic infection in carp [20]. Both serve as valuable indicators for evaluating dietary iron supplementation in this study.

Tomatoes are highly adaptable to environmental changes, with a root system that is well suited for hydroponic systems, allowing for efficient nutrient absorption from the water. In hydroponic systems, tomatoes can be cultivated vertically or in compact spaces, thereby significantly enhancing yield per unit area [21]. Furthermore, under aquaponic conditions, tomatoes exhibit improved disease resistance, even in the absence of pesticides [22]. Pak choi, with its short growth cycle and strong adaptability, thrives in a variety of hydroponic systems. It has relatively low demands for temperature and water quality, making it particularly well suited for hydroponic systems [23]. Based on these characteristics, tomatoes and pak choi were chosen as the experimental vegetables for this study.

Ferric EDTA, or ferric sodium ethylenediaminetetraacetate, is a chelated form of iron with the chemical formula C_10_H_12_FeN_2_NaO_8_. Commonly found as a yellowish-brown powder, it is used as a foliar spray in agriculture or as a dietary supplement. Without proper chelating agents, ferric ions in food precipitate at pH levels above 3.5, limiting absorption. Studies confirm that ferric EDTA facilitates iron absorption by physiological pathways, as iron dissociates from the chelate before absorption. Compared to inorganic iron solutions, ferric EDTA exhibits superior performance as a food additive [24,25].

In summary, fish primarily acquire iron through dietary intake, with transferrin transporting and storing iron in the liver. Hepcidin regulates iron uptake, which directly affects growth, hematopoiesis, and immunity. However, the optimal amount of iron released into the system by fish in aquaponic systems has not yet been determined. This study aims to determine the optimal level of iron supplementation in feed by evaluating plant growth parameters, the growth of mirror carp (*Cyprinus carpio var. specularis*), hematological and immune parameters, as well as hepatopancreatic condition to simultaneously meet the growth requirements of mirror carp and ensure that the iron released into the system through carp metabolism is sufficient for plant growth. Ferric EDTA is used as the iron source in this study to address the iron requirements in aquaponic systems.

## 2. Materials and Methods

### 2.1. Experimental Design

The 90-day experiment was conducted in a plastic greenhouse at the Taigu campus of Shanxi Agricultural University, covered with a 50% light-transmitting shade net. Twelve wooden fish tanks (90 cm length × 60 cm width × 70 cm height) were used, with tanks 1–6 arranged in a row on the eastern side and tanks 7–12 on the western side of the greenhouse. To balance light exposure, tanks 1, 6, and 7 were set as the control group (T0); tanks 2, 3, and 12 formed experimental group T1; tanks 4, 8, and 9 formed experimental group T2; and tanks 5, 10, and 11 formed experimental group T3.

**Figure 1:**
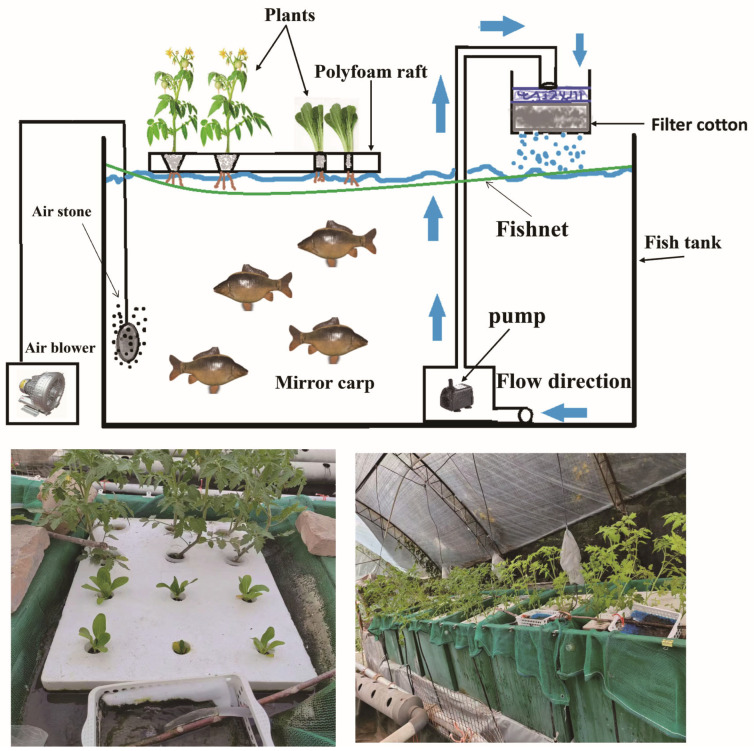
Schematic diagram of the floating valve-based aquaponic system.

Each tank was equipped with a water pump connected to a mat of vine cotton placed over a filter cloth, both housed in a plastic basket fixed above the tank. Filtered water flowed back into the tanks. An air pump with multi-porous aeration tubes supplied oxygen to all tanks. The water level was marked with a marker for monitoring, and water was added daily to maintain consistent tank volumes. Filters and vine cotton were not cleaned during the experiment to ensure a “no water change” and “no fertilizer” aquaponics concept. Each tank was fitted with a foam board (60 cm × 45 cm), perforated with six large holes and six small holes for planting. Tomato seedlings, sourced from the Taigu Juxin Agricultural Base, were 20.43 ± 1.25 cm in height, with a stem diameter of 3.24 ± 0.21 mm and 28.33 ± 3.27 leaves. Each large hole housed one tomato (*Solanum lycopersicum*) seedling secured in planting cotton. Pak choi (*Brassica rapa* subsp. *chinensis*) seeds were pre-germinated for seven days in damp planting cotton, then placed in the smaller holes. Daily foliar spraying prevented pest damage. A loose plastic mesh was fixed above each tank to prevent fish from damaging roots or jumping out. Foam boards floated on the water, ensuring all planting cotton was in contact with the water (Figure 1). The experiment used 240 uniformly sized mirror carp (*Cyprinus carpio var. specularis*) (average length: 9.98 ± 0.47 cm; average weight: 19.66 ± 2.37 g), with 20 fish per tank. Temperature, pH, and dissolved oxygen (DO) levels were measured every 15 days using a thermometer, pH meter, and DO meter.

### 2.2. Feeding Method and Feed Preparation

Commercial 2 mm extruded feed was used. Ferric EDTA of analytical grade was dissolved in 100 mL distilled water, then evenly sprayed onto the feed and air-dried at room temperature for 12 h. Sodium alginate (≥99% purity) was dissolved in distilled water at a content of 2% (*w*/*v*) and sprayed onto the feed to form a protective coating, preventing the ferric EDTA from dissolving in the water. Only sodium alginate was applied for the control group, T0. Feed nutritional content was analyzed, and feed for each group was weighed before feeding. Fish were fed twice daily, with a feed amount equivalent to 1% of the total body weight in the tank per feeding. A fixed sound signal was used during feeding to condition the fish. Observations ensured that all feed was consumed within 20 min, leaving no residuals in the tank. The nutritional composition of the commercial feed was as follows: crude protein content 31.76 ± 9.41%, crude fat content 6.67 ± 0.55%, moisture content 15.24 ± 0.96%, and ash content 10.31 ± 0.21%. Regarding trace elements, the iron content was 337.35 ± 9.41 mg/kg, and the copper content was 56.8 ± 1.61 mg/kg. Additionally, the iron (Fe) content in the experimental feed groups was as follows: T0 group, 337.35 ± 9.41 mg/kg; T1 group, 514.22 ± 19.74 mg/kg; T2 group, 752.80 ± 31.82 mg/kg; and T3 group, 1178.81 ± 39.98 mg/kg (Table 1).

### 2.3. Sampling and Measurement of Vegetables and Fish

Tomatoes: Plant height (cm): measured from the root collar to the growth tip using a measuring tape. Stem diameter (mm): measured 2 cm above the root collar using calipers. Leaf count: total number of fully expanded leaves manually counted. On Days 60 and 90, the number of flowers and fruits were recorded: Flower count: number of fully open flowers per plant. Fruit count: number of formed fruits per plant. Pak Choi: harvested on Day 60, fresh weight (g) was measured using an electronic balance.

Every 30 days, four fish per tank were randomly sampled for length and weight measurements. The fish were anesthetized by placing them in an MS222 solution (25 g/m^3^, and measurements were taken once the fish no longer responded to stimuli. After measurements, the fish were immediately dissected. Blood was collected from the tail vein using a 1 mL syringe pre-washed with 2.7% EDTA and transferred to anticoagulant tubes. Blood was gently mixed and split into two parts: one part for red blood cell counts and hemoglobin content measurement, stored at 4 °C and analyzed on the same day, the other for serum separation via centrifugation (3500 rpm, 15 min, 4 °C). Serum was stored in sterile cryovials at −80 °C for subsequent analysis.

Fish were fasted for 24 h prior to health parameter analysis. The visceral mass was extracted, and the liver was divided into two parts: one for enzyme activity measurement at −80 °C and the other for histological sectioning.

Growth Performance Metrics:

The growth parameters of mirror carp were calculated based on sampling data from days 0–30, 31–60, and 61–90. Specifically, the initial weight on day 60 was the final weight recorded on day 30, and the initial weight on day 90 was the final weight recorded on day 60.

Weight Gain Rate (WGR, %) = (Final Weight (g) − Initial Weight (g))/Initial Weight (g) × 100%(1)

Specific Growth Rate (SGR, %) = [ln (Final Weight (g)) − ln (Initial Weight (g))]/Experiment Days × 100%(2)

Condition factor (CF, g/cm^3^) = Weight of fish (g)/{body length of fish(cm)}^3^ (3)

Protein Efficiency Ratio (PER, %) = Weight Gain (g)/Crude Protein Intake (g) × 100%(4)

Feed Conversion Ratio (FCR) = Total Feed Intake (g)/(Final Weight (g) − Initial Weight (g))(5)

### 2.4. Determination of Fish Nutritional Composition

The contents of moisture, crude protein, crude fat, and ash in fish samples were analyzed according to standard laboratory protocols (AOAC). Crude protein was measured using the Kjeldahl nitrogen determination method [26].Crude fat was determined by the Soxhlet extraction method [27]. Moisture content and ash content were evaluated following standard procedures [28,29].

### 2.5. Determination of Elemental Content in Water and Mirror Carp (Cyprinus carpio var. specularis) Liver

Elemental content in liver and water samples was quantified using an inductively coupled plasma optical emission spectrometer (ICP-OES) manufactured by Shimadzu, Kyoto, Japan. Samples were prepared using microwave digestion [30].

### 2.6. Processing of Blood and Tissue Samples

Red blood cell count was performed using a hemocytometer. Hemoglobin content, serum SOD activity, serum lysozyme activity, serum transferrin levels, and serum hepcidin levels were determined using commercial kits provided by Nanjing Jiancheng Bioengineering Institute, Nanjing, China. Hepatic SOD activity and CAT activity were also assessed using these kits.

### 2.7. Tissue Sectioning

Liver tissue fragments were fixed in 4% paraformaldehyde solution, embedded in paraffin, sectioned into 5 μm slices, and mounted on glass slides. After dehydration, the sections were stained with hematoxylin and eosin (H&E) to observe histological changes. Image analysis and measurement were performed using IMAGE J 1.5.3 software.

### 2.8. Data Analysis Methods

All data are expressed as mean ± S.E.M. One-way analysis of variance (ANOVA) was performed using SPSS 26.0 (IBM) for Windows to determine significance levels. Duncan’s multiple range test was employed for multiple comparisons. Statistical significance was set at *p* < 0.05. Graphs were created using GraphPad Prism 6.0 (GraphPad Software, San Diego, CA, USA).

## 3. Results

### 3.1. Temperature, Dissolved Oxygen (DO), pH, and Iron Content

**Figure 2:**
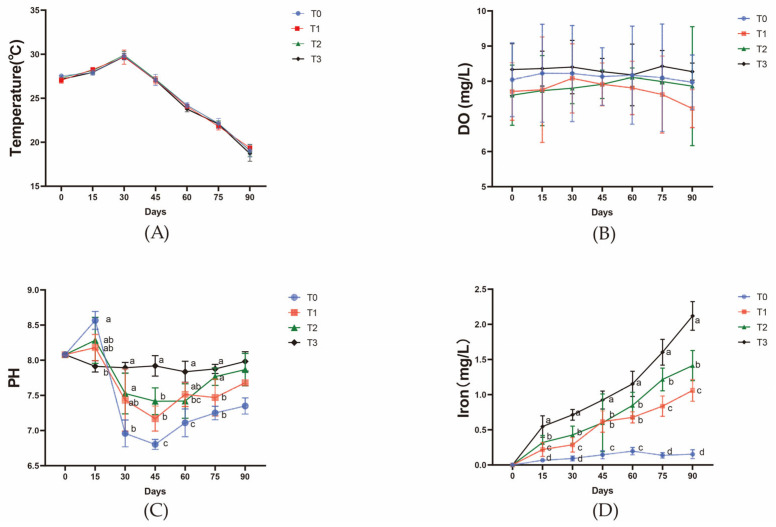
Water quality parameters. (**A**) Water temperature in each group of fish tanks; (**B**) dissolved oxygen levels in each group of fish tanks; (**C**) pH values of the water in each group of fish tanks; (**D**) iron content in the water of each group of fish tanks. The data presented with various superscripts such as a, b, and c reflect significant differences (*p* < 0.05) (*n* = 3).

Throughout the experiment, the water temperature and dissolved oxygen (DO) content remained within the optimal range for the growth of both tomatoes and pak choy, with stable temperatures between 18 and 30 °C, and DO levels consistently above 7 mg/L. The addition of ferric EDTA to the feed significantly increased the iron content in the water, with the highest content (1.8 mg/L) observed in the T3 group (800 mg/kg iron). Water pH fluctuated noticeably throughout the experiment. During the first 15 days, the pH in all groups decreased slightly, with a more pronounced decrease in the control group (T0) (*p* < 0.05). From days 15 to 60, all treatment groups exhibited a stabilizing trend in pH. Group T3 maintained a stable pH range of 7.2–7.5, while groups T0 and T1 had a lower pH range (6.8–7.2). After day 60, the pH of T0 and T1 increased slightly (7.4–7.6), whereas T2 and T3 groups remained at a relatively lower pH range (6.8–7.0). Group T0 had a significantly lower pH compared to the other groups (*p* < 0.05) (Figure 2).

### 3.2. Growth of Tomatoes and Bok Choy

Table 2 shows that after 30 days of feeding with varying iron content, the tomato plants in groups T1, T2, and T3 exhibited significantly greater plant height and stem diameter compared to the control group (T0) (*p* < 0.05). Among the experimental groups, T3 plants showed the highest growth, followed by T1 and T2. No significant differences were found in leaf number and flower count.

At day 60 (Table 3), the results showed that T2 plants had significantly higher plant height and stem diameter than T1, although there were no significant differences between T1 and T3. The fresh weight of pak choy harvested from T1, T2, and T3 was significantly higher than that from the control group (*p* < 0.05). Pak choy from group T3 had the highest fresh weight, significantly greater than T1 and T2. Fruit set in tomatoes showed no significant differences among groups, but flower number and fruit count were significantly higher in T2 and T3 compared to T0 (*p* < 0.05).

At day 90 (Table 4), the tomato plants’ height and stem diameter remained consistent with the previous measurements at day 60. The fruit set was significantly higher in the experimental groups (T1, T2, and T3) compared to the control (*p* < 0.05), though there were no significant differences between the experimental groups. No significant differences in flower count were observed.

### 3.3. Growth and Nutritional Composition of Mirror Carp (Cyprinus carpio var. specularis)

During the first 60 days, no significant differences in growth or nutritional composition were observed among the groups (Table 5 and Table 6). However, at day 90 (Table 7), the weight, weight gain, specific growth rate (SGR), protein efficiency ratio (PER), and feed conversion ratio (FCR) of the T3 group (800 mg/kg iron) were significantly lower than those of the T0, T1, and T2 groups (*p* < 0.05). Nutritionally, the crude fat content in the T2 and T3 groups was significantly lower than in the T1 group (*p* < 0.05), while no significant differences were observed between T0 and the other three groups.

### 3.4. Blood Parameters and Enzyme Activity in Mirror Carp (Cyprinus carpio var. specularis)

**Figure 3:**
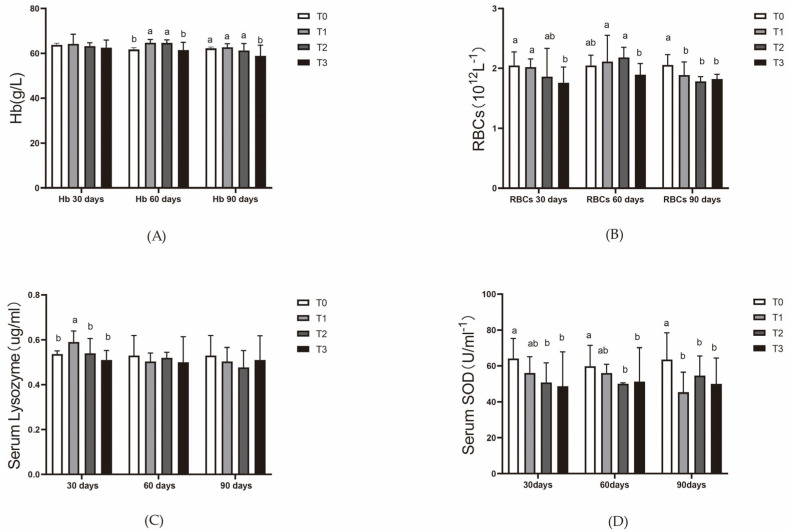
Effect of dietary ferric EDTA levels in aquaponic system on hematological parameters and enzyme activities in serum of mirror carp. (**A**) Red blood cell count; (**B**) hemoglobin content; (**C**) serum lysozyme activity; (**D**) serum SOD (superoxide dismutase) enzyme activity. The data presented with various superscripts such as a and b reflect significant differences (*p* < 0.05) (*n* = 3).

At day 30, red blood cell counts in the T3 group were significantly lower than in T0 and T1 (*p* < 0.05), with no significant difference compared to T2. At day 60, red blood cell counts in T3 were significantly lower than in T1 and T2 (*p* < 0.05), but no significant difference was observed compared to T0. By day 90, hemoglobin content at day 60 was significantly higher in T1 and T2 compared to T0 and T3 (*p* < 0.05), and at day 90, T3 had significantly higher hemoglobin levels than the other groups (*p* < 0.05) (Figure 3A), the red blood cell count in T0 was significantly higher than in the other groups (*p* < 0.05), and no significant differences were observed among T1, T2, and T3 (Figure 3B).

At day 30, the serum lysozyme content in T1 was significantly higher than that in the other three groups (*p* < 0.05). There were no significant differences in serum lysozyme levels at day 60 and day 90 (Figure 3C).

At day 30, the serum SOD enzyme activity in T0 was significantly higher than in T2 and T3, with no significant differences among the T1, T2, and T3 groups. The pattern at day 60 was similar to that at day 30. At day 90, the SOD enzyme activity in T0 was significantly higher than in the other three groups (*p* < 0.05) (Figure 3D).

**Figure 4:**
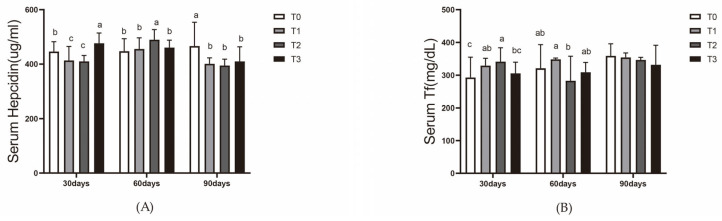
Effect of dietary ferric EDTA levels in aquaponic system on hepcidin level and transferrin level in blood of mirror carp. (**A**) Serum transferrin content; (**B**) serum hepcidin content. The data presented with various superscripts such as a, b, and c reflect significant differences (*p* < 0.05) (*n* = 3).

At day 30, transferrin levels in T1 and T2 were significantly higher than those in T0 and T3 (*p* < 0.05). At day 60, T1 exhibited the highest transferrin levels, significantly surpassing those in the other groups (*p* < 0.05). Transferrin levels in T3 were the lowest but not significantly different from those in T2. At day 90, there were no significant differences in transferrin levels among the groups, but T3 still maintained the lowest level (Figure 4A).

At day 30, the hepcidin level in T3 was significantly higher than that in T0 (*p* < 0.05), while T0 exhibited significantly higher levels than T1 and T2 (*p* < 0.05). At Day 60, the hepcidin level in T2 was significantly higher than in the other three groups, with no significant differences among the other groups. At day 90, the hepcidin level in T0 was significantly higher than in the other three groups, which showed no significant differences among themselves (Figure 4B).

### 3.5. Effects on Liver Tissue

**Figure 5:**
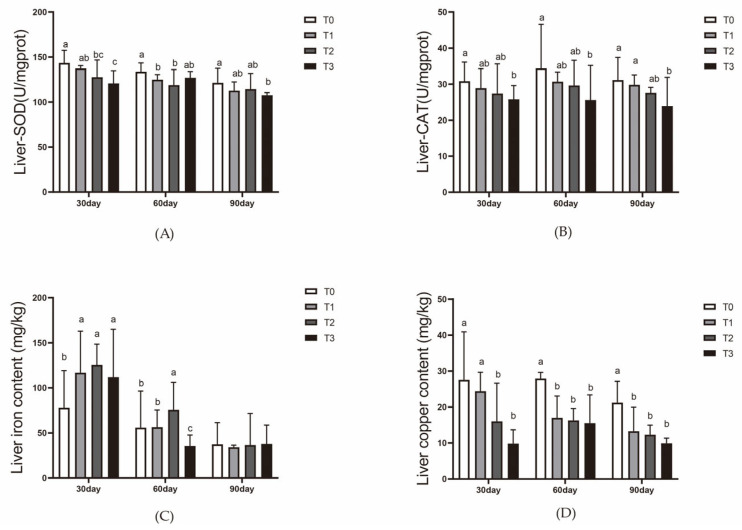
Effect of dietary ferric EDTA levels in aquaponic system on liver antioxidant indexes of mirror carp. (**A**) Liver SOD enzyme activity; (**B**) liver CAT (catalase) enzyme activity; (**C**) liver iron content; (**D**) liver copper content. The data presented with various superscripts such as a, b, and c reflect significant differences (*p* < 0.05) (*n* = 3).

At day 30, SOD activity in the liver was significantly higher in T0 compared to the T1, T2, and T3 groups (*p* < 0.05), with the T3 group (high iron content) showing the lowest SOD activity. At days 60 and 90, SOD activity was significantly lower in all experimental groups (T1, T2, and T3) compared to T0 (*p* < 0.05). (Figure 5A).

**Figure 6:**
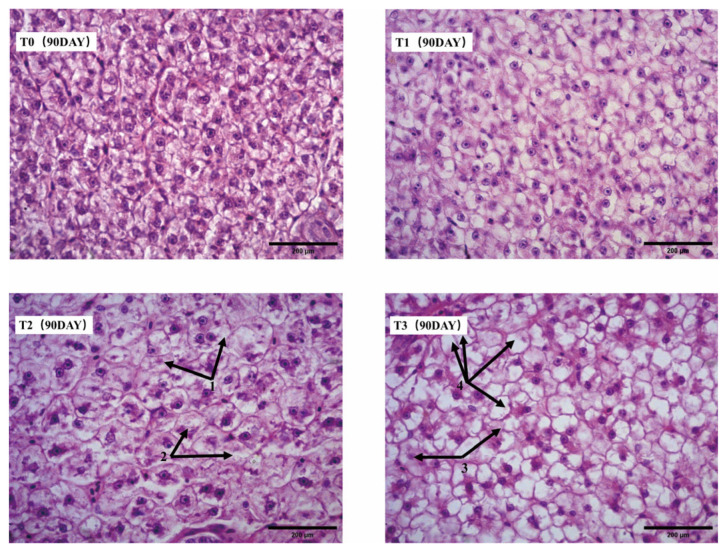
Liver tissue sections of mirror carp *(Cyprinus carpio var. specularis)* at day 90 for all groups. (1) Mild cloudy swelling was observed in some hepatocytes, with indistinct cellular contours. (2) Partial nuclear fragmentation and dissolution were evident in affected cells. (3) A significant level of nuclear dissolution was observed in hepatocytes. (4) Hepatocytes exhibited severe vacuolization, indicating extensive cellular damage.

After 90 days, hepatocytes in the T0 and T1 groups exhibited no significant pathological changes. In the T2 group, some hepatocytes showed hydropic degeneration with blurred cellular contours, and certain nuclei displayed karyorrhexis and karyolysis. Pathological changes were more pronounced in the T3 group, where extensive karyolysis was observed alongside severe hepatocyte vacuolation (Figure 6).

Liver iron content was significantly higher in T2 at day 30 compared to the other groups (*p* < 0.05). At days 60 and 90, the iron content in the liver decreased slightly in all groups, reaching a balanced level by day 90 (Figure 5C). The copper content in the liver was significantly higher in the experimental groups (T1, T2, and T3) at day 30 compared to T0 (*p* < 0.05), with T1 and T2 showing similar levels, while T3 had slightly higher copper content. At days 60 and 90, the copper content remained higher in T1, T2, and T3 compared to T0 (*p* < 0.05) (Figure 5D).

### 3.6. The Correlation Between Dietary Iron Supplementation and Iron Content in Water and the Correlation Between Mirror Carp Weight Gain and Vegetable Growth

**Figure 7:**
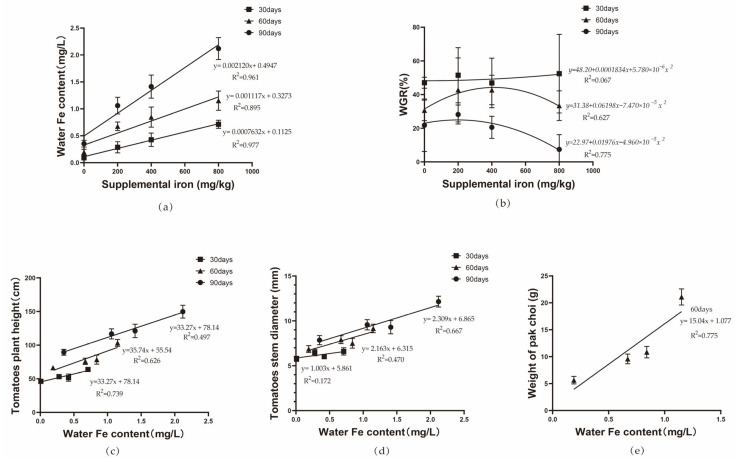
The correlation between dietary iron supplementation and iron content in water; the correlation between mirror carp weight gain and vegetable growth. (**a**) Regression analysis between supplemental iron levels in the diet and iron content in water. (**b**) Regression analysis between supplemental iron levels in the diet and weight gain rate of mirror carp. (**c**) Regression analysis between iron content in water and tomatoes plant height. (**d**) Regression analysis between iron content in water and tomatoes stem diameter. (**e**) Regression analysis between iron content in water and pak choy weight.

The regression analysis between supplemental iron levels in the diet and iron content in water, shown in Figure 7a, reveals a linear positive correlation between water iron content and dietary iron supplementation on days 30, 60, and 90. According to the quadratic regression analysis in Figure 7b, the optimal weight gain rate of mirror carp is achieved when 400 mg/kg of ferric EDTA is added to the diet on day 60 and 200 mg/kg on day 90. Furthermore, the regression curves in Figure 7c–e show that the growth of vegetables is linearly positively correlated with iron content in water.

## 4. Discussion

### 4.1. Effects of Iron Supplementation in Diet on Water Quality and Vegetable Growth

Iron plays a critical role in plant photosynthesis, pigment production, and protein synthesis. In aquaponic systems, plants frequently suffer from iron deficiencies, as the iron content in fish excreta is generally inadequate to support their growth requirements [31]. In this experiment, the temperature and dissolved oxygen levels in the water were maintained within optimal ranges for the growth of tomatoes and pak choi (*Brassica rapa* subsp. *chinensis*). As the feeding duration increased, the iron content in the water also rose. Regression analysis indicated a linear positive correlation between the two. After the pak choi was harvested on day 60, there were no significant changes in water quality. Roosta et al. found that hydroponically grown tomatoes (*Solanum lycopersicum*) thrive best when the nutrient solution contains 1.1 mg/L of iron and the water pH is between 7.0 and 7.7 [2]. Additionally, studies have shown that adequate iron supplementation promotes faster growth rates and improved nutritional quality in hydroponically grown pak choi (*Brassica rapa* subsp. *chinensis*) [32]. In this study, plant height, stem diameter, leaf count of tomatoes measured between days 30 and 90, fruit count on day 90, and the fresh weight of pak choi (*Brassica rapa* subsp. *chinensis*) harvested at day 60 all showed a significant positive correlation with the iron content in the water. These findings suggest that iron supplementation had a beneficial impact on plant growth. One reported strategy for iron uptake in higher plants involves root acidification, where roots release hydrogen ions to facilitate iron absorption, a process that consumes energy [33]. Consequently, groups with lower iron supplementation exhibited a reduction in water pH, which may explain the improved plant growth in the high-iron supplementation groups. Research by Kwong et al. on rainbow trout *(Oncorhynchus mykiss)* suggested that iron absorption in the anterior intestine may occur via simple diffusion, while carrier-mediated pathways predominate in the middle and posterior intestines. Notably, an increase in intestinal mucosal pH from 7.4 to 8.2 significantly reduced iron absorption in the mid and hindgut [34]. Tyson et al. highlighted that maintaining the water pH between 7.5 and 8.0 can enhance sustainable productivity in aquaponic systems [35]. In this experiment, the water pH fluctuated between 6.8 and 8.5, remaining conducive to the sustainable development of aquaponics systems.

### 4.2. Effects of Iron Supplementation in Diet on Blood and Immune Enzyme Activity in Mirror Carp (Cyprinus carpio var. specularis)

Iron plays a critical role in erythropoiesis and hemoglobin synthesis [36]. Zhang et al. reported that both deficiency and excess dietary iron reduce red blood cell (RBC) counts in grass carp (*Ctenopharyngodon idellus*) [37]. Similarly, Musharraf et al. [38] demonstrated that supplementing diets for Indian carp *Labeo rohita* (Hamilton) with 13.2–427.3 mg/kg of iron led to increased RBC counts and hemoglobin levels up to 218.8 mg/kg; however, further increases in dietary iron (up to 427.3 mg/kg) did not result in significant differences. Additionally, research indicates that RBC production correlates with serum hepcidin levels. Elevated hepcidin can suppress the release of recycled RBCs into the bloodstream [39]. Pasricha et al. [40] further elucidated the mechanisms underlying the interplay between RBC production and hepcidin regulation. In this study, RBC counts corresponded to serum hepcidin levels at day 30. Groups with higher serum hepcidin exhibited lower RBC counts, likely due to the inhibitory effect of elevated hepcidin on erythropoiesis in mirror carp (*Cyprinus carpio var. specularis*). At days 60 and 90, groups receiving higher dietary iron showed reduced RBC counts, possibly due to decreased hepatic iron content impairing hematopoiesis. Pan et al. [41] also observed that hepatic iron levels influence RBC counts, with lower hepatic iron content associated with decreased RBC production. Hemoglobin is composed of iron atoms and heme groups; insufficient iron intake impairs hemoglobin synthesis [42]. Shiau and Su [43] found that increased dietary iron enhanced hemoglobin synthesis and RBC counts in tilapia (*Oreochromis niloticus* × *O. aureus*). At day 60, the T1 and T2 groups exhibited significant increases in hemoglobin levels, suggesting that iron supplementation enhanced hematopoiesis. Conversely, hemoglobin levels in the T3 group were significantly lower than in other groups, potentially due to the suppressive effects of hepcidin on erythropoiesis.

Studies have shown that inorganic mineral supplementation can elevate serum lysozyme and superoxide dismutase (SOD) activities [44,45]. Hermes et al. [46] found that organic iron supplementation resulted in the lowest lysozyme activity among Nile tilapia *(Oreochromis niloticus)*. Similarly, Valenzuela-Muñoz et al. reported that iron overload downregulated immune-related genes in Atlantic salmon (*Salmo salar*). In this study, lysozyme activity was higher in the T1 group at day 30, while no significant differences were observed at days 60 and 90. This suggests that initial ferric EDTA supplementation boosted immunity in mirror carp (*Cyprinus carpio var. specularis*), but levels normalized as fish adapted. Studies by Mao et al. [47] and revealed that dietary iron supplementation increased serum SOD activity in largemouth bass (*Micropterus salmoides*) and coho salmon *Oncorhynchus kisutch*, respectively. However, in this study, serum SOD activity in the T1, T2, and T3 groups was significantly lower than in the control group, potentially due to elevated serum iron levels suppressing SOD activity. Research by Xiao et al. [48] showed that excessive iron intake in humans reduced serum SOD activity due to increased serum iron content.

### 4.3. Effects of Iron Supplementation in Diet on Serum Transferrin, Hepcidin, and Serum Biochemicals in Mirror Carp (Cyprinus carpio var. specularis)

Hepcidin regulation mechanisms in fish closely resemble those in mammals and are conserved among teleost [49]. The liver synthesizes hepcidin, which regulates dietary iron absorption, iron recycling by splenic and hepatic macrophages, and the release of stored iron from hepatocytes via ferroportin (FPN). Under iron-deficient conditions, hepcidin synthesis decreases, facilitating FPN-mediated iron transport into cells. Conversely, iron overload stimulates hepcidin production, which binds to FPN, reducing iron uptake and storage [18,50]. In bighead carp (*Aristichthys nobilis*), dietary supplementation with FeSO_4_ for 60 days demonstrated that serum hepcidin levels decreased with dietary iron up to 162 mg/kg, but increased at 203.1 mg/kg [51]. In this study, hepcidin levels were significantly lower in the T1 and T2 groups than in the control group (T0) at day 30, while the T3 group showed elevated levels. By day 60, hepcidin levels in the T2 group were significantly higher than in other groups. At day 90, serum hepcidin levels in the control group exceeded those in all experimental groups. These results suggest that at day 30, the T1 and T2 groups reduced hepcidin synthesis to enhance iron uptake and storage, while the T3 group reached iron saturation, leading to elevated hepcidin synthesis. By day 60, iron storage in the T1 and T2 groups was saturated, increasing plasma hepcidin levels. At day 90, reduced erythropoiesis from excessive iron intake triggered feedback regulation of hepcidin [52]. Transferrin (Tf), synthesized in the liver and secreted into the bloodstream, efficiently binds ferric iron for transport to various cells. Serum transferrin content is an important indicator of systemic iron balance [53,54]. In this study, serum transferrin levels corresponded inversely to hepcidin levels at days 30 and 60, indicating their coordinated role in iron metabolism. When hepcidin decreased, transferrin increased to facilitate iron transport, and vice versa. Elevated hepcidin may suppress transferrin synthesis [55].

### 4.4. Effects of Iron-Enriched Feed on the Liver of Mirror Carp (Cyprinus carpio var. specularis)

The liver serves as the primary organ for iron storage in fish. Acute iron stress has been reported to cause hepatic cell damage in Rohu *Labeo rohita* [56]. A study by Buyinza et al. revealed that feeding channel catfish (*Ictalurus punctatus*) with 250 mg/kg FeSO_4_-enriched diets for 10 weeks resulted in iron overload, leading to hepatic cell damage and even apoptosis [57]. Similarly, in this study, at day 90, the livers of mirror carp *(Cyprinus carpio var. specularis)* in the T2 and T3 groups exhibited pathological changes in liver cells, including cloudy swelling, unclear cell boundaries, extensive nuclear dissolution, and severe vacuolation. These results indicate inflammatory changes, suggesting that excessive dietary iron in the T2 and T3 groups caused liver tissue damage in mirror carp *(Cyprinus carpio var. specularis)*.

Fish counteract oxidative stress induced by elevated reactive oxygen species (ROS) by increasing the activity of antioxidative enzymes like superoxide dismutase (SOD) and catalase (CAT), thereby reducing oxidative cellular damage [58,59,60]. Previous studies demonstrated that higher dietary iron levels enhance antioxidative enzyme activity on Cyprinus and *Labeo rohita* [38,61]. However, in this study, SOD and CAT activities in the liver showed significant decreases. On day 30, SOD activity in the T2 and T3 groups was significantly lower than in the control group (T0), with T3 also being significantly lower than T1. Similarly, CAT activity in T3 was markedly lower than in T0. It is hypothesized that the high dietary iron intake in the experimental groups triggered the Fenton reaction in the liver, exacerbating ROS production and subsequently reducing SOD and CAT activity [62]. The trends persisted at days 60 and 90, where SOD activity in T1 and T2 was lower than in T0, and both enzymes exhibited the lowest activity in the high-iron T3 group. Based on liver histology at day 90, long-term iron intake likely caused cellular damage, leading to decreased antioxidative enzyme activity. Luo et al. reported that excessive iron supplementation in aquaponics systems suppressed SOD and CAT activities in the livers of mirror carp *(Cyprinus carpio var. specularis)* [63]. Singh et al. similarly found that increased water iron levels reduced SOD and CAT activity in *Labeo rohita*, accompanied by varying degrees of hepatic cell damage [56].

Research on dietary iron supplementation in mirror carp (*Cyprinus carpio var. specularis*) and Rohu (*Labeo rohita*) has shown that hepatic iron levels rise with increased dietary iron [38,64]. However, a study by Pan et al. demonstrated that when dietary iron levels for juvenile gibel carp (*Carassius auratus gibelio*) increased from 138 mg/kg to 223 mg/kg, hepatic iron content rose correspondingly. Beyond 223 mg/kg, up to 273 mg/kg, hepatic iron content plateaued [41]. Similar trends were observed in coho salmon (*Oncorhynchus kisutch*) fed FeSO_4_-enriched diets [65]. In this study, at day 30, hepatic iron levels in T2 were significantly higher than in T1, and T1 was higher than in T0 and T3. However, by days 60 and 90, hepatic iron levels showed no significant differences among the four groups. It is speculated that by day 60, T3 had reached the hepatic iron saturation threshold, triggering mechanisms to reduce iron storage. At day 90, the cumulative dietary and waterborne iron caused all groups to reach saturation. The decline in hepatic iron at this stage may be attributed to hepcidin regulation, which limits hepatic iron storage via various pathways [66]. Copper metabolism parallels iron metabolism, involving intestinal absorption, blood transport, and hepatic storage. Both metals utilize the DMT1 transporter for cellular uptake [13]. Excessive iron intake can lead to hepcidin-mediated degradation of DMT1, reducing its levels [67]. Additionally, research by Kwong et al. highlighted that intestinal pH influences iron transporter activity [34]. In this study, hepatic copper levels in the iron-supplemented groups (T1, T2, and T3) were significantly higher than in T0 throughout the 90-day period. This phenomenon may result from dietary iron reducing DMT1 levels in the intestines of mirror carp *(Cyprinus carpio var. specularis)*, indirectly affecting copper metabolism.

### 4.5. Effects of Iron Supplementation in Feed on the Growth and Nutritional Content of Mirror Carp (Cyprinus carpio var. specularis)

In this 90-day experiment, no fish mortality was observed. During the first 60 days, there were no significant differences in the growth performance and nutritional composition among the mirror carp (*Cyprinus carpio var. specularis*) in all groups. However, by day 90, fish in the T3 group, which received a feed supplemented with 800 mg/kg ferric EDTA, exhibited significant reductions in all growth parameters, including body weight, feed conversion ratio, and specific growth rate. Building on the previous analysis of water quality and plant growth within the system, it is evident that high iron content in the feed have a direct impact on the iron released into the water through the metabolic processes of mirror carp. This, in turn, affects plant growth and induces alterations in water pH, which subsequently influences the growth of mirror carp. Additionally, the biochemical analysis of mirror carp reveals that excessive iron supplementation leads to elevated serum hepcidin levels, which consequently reduce hepatic iron storage capacity, induce pathological changes in the liver, and decrease red blood cell count. Furthermore, high iron intake results in the suppression of antioxidant enzyme activity in both the blood and hepatopancreas. These factors are inferred to contribute to the observed decline in mirror carp performance. Conversely, the addition of an optimal iron content in the feed appears to improve these parameters, thereby promoting optimal growth performance in mirror carp. Previous research on dietary iron supplementation in mirror carp (*Cyprinus carpio var. specularis*) demonstrated significant improvements in growth performance and nutritional status when ferrous sulfate was included at levels between 118.22 and 120.44 mg/kg [64]. Studies have shown that the optimal growth temperature for mirror carp is 25–30 °C [68]. However, in this experiment, the lowest temperature in the greenhouse on day 90 was 18 °C, which may explain why the growth coefficients of mirror carp in all experimental groups were lower on day 90 compared to days 30 and 60. Regarding nutritional composition, crude fat levels in the T2 and T3 groups were significantly lower than in the T1 group, while other parameters showed no significant differences. Additionally, the study found that dietary iron supplementation reduced triglyceride levels and affected fatty acid synthesis in coho salmon (*Oncorhynchus kisutch*) [69].

## 5. Conclusions

In this 90-day experiment, the addition of ferric EDTA to the feed significantly promoted the growth of vegetables (tomatoes and pak choi) in the aquaponic system. Adequate iron supplementation also mitigated the effects of root acidification caused by iron deficiency, which reduces the impact of lowered pH on fish. On day 30, the iron content in the liver of the mirror carp (*Cyprinus carpio var. specularis*) in the T1 (200 mg/kg) and T2 (400 mg/kg) groups reached its peak, while liver copper levels were consistently lower in the experimental groups than in the control group throughout the 90-day period. After 60 days of feeding, the T1 and T2 groups showed enhanced hematopoiesis and improved immunity in the mirror carp. However, by day 90, slight degeneration of hepatopancreatic cells was observed in the T2 group, while the T3 group, fed with 800 mg/kg ferric EDTA, exhibited growth suppression and liver and pancreas tissue damage.

Overall, these results suggest that the T2 group—which received 200 mg/kg of EDTA iron in the feed, with a total iron content of 514.22 mg/kg in the feed over 60 days in a non-water-changing aquaponic system—produced optimal fish and vegetable yield. These findings provide valuable insights for optimizing ferric EDTA supplementation in floating valve-based aquaponic systems.

## Figures and Tables

**Table 1:** Formulation and proximate composition of the experimental diets (expressed as % dry matter).

Ingredients (%)	
Fish meal	21
Soybean meal	23
Wheat flour	21
Soy oil	4
Fish oil	3
NaCl	1
Corn starch	25
Vitamin premix ^1^	1
Mineral premix ^2^	1
Proximate composition	
Moisture content (%)	15.24
Ash content (%)	10.31
Crude protein (%)	31.76
Crude fat (%)	6.67
Total iron content (mg/kg)	337.35
Total copper content (mg/kg)	24.8

^1^ Per kilogram of vitamin premix containing vitamin A, 500,000 IU; vitamin D3, 1,000,000 IU; vitaminK3, 3.5 g; vitamin E, 16 g; vitamin B1, 3.5 g; vitamin B2, 7 g; vitamin B6, 7 g; vitamin B12, 0.001 mg; vitamin C, 35 g; biotin, 0.01 g; folic acid, 0.2 g; niacin, 35 g; Ca-D-pantothenate, 16 g; inositol, 35 g. ^2^ Per kilogram of mineral premix containing (g/kg mixture) Ca(H_2_PO_4_)_2_, 155 g; FeSO_4_·7H_2_O, 150 g; MgSO_4_·7H_2_O, 100 g; AlCl_3_·6H_2_O, 0.6 g; MnSO_4_·H_2_O, 4 g; KCl, 40 g; CuSO_4_·5H_2_O, 10 g; KI, 0.6 g; CoCl_2_·6H_2_O, 2 g; ZnSO_4_·7H_2_O, 20 g.

**Table 2:** Growth performance during a planting period of 30 days.

30 Days	Plant Height (cm)	Stem Diameter (mm)	Leaf Count	Flower Count
T0	46.14 ± 5.95 ^c^	5.77 ± 0.45 ^b^	104.16 ± 19.3	0.16 ± 0.57
T1	53.15 ± 5.20 ^b^	6.47 ± 0.58 ^a^	108.00 ± 20.77	0.25 ± 0.45
T2	51.68 ± 8.41 ^b^	6.01 ± 0.42 ^b^	104.25 ± 20.82	0.08 ± 0.28
T3	63.70 ± 2.66 ^a^	6.60 ± 0.63 ^a^	107.66 ± 25.27	0.00 ± 0

The data presented with various superscripts such as a, b, and c reflect significant differences (*p* < 0.05) (*n* = 4).

**Table 3:** Growth performance during a planting period of 60 days.

60 Days	Plant Height (cm)	Stem Diameter (mm)	Flower Count	Fruit Count	Pak Choi Fresh Weight (g)
T0	66.41 ± 5.33 ^c^	6.85 ± 0.60 ^c^	2.08 ± 1.50	0.58 ± 0.66	5.64 ± 1.09 ^c^
T1	75.92 ± 7.33 ^b^	7.90 ± 0.68 ^b^	2.91 ± 1.97	1.25 ± 1.13	9.58 ± 1.42 ^b^
T2	78.42 ± 10.78 ^b^	7.50 ± 0.86 ^b^	3.25 ± 2.13	1.00 ± 0.85	10.83 ± 1.65 ^b^
T3	103.25 ± 8.17 ^a^	9.15 ± 0.75 ^a^	2.91 ± 2.02	1.00 ± 1.20	21.09 ± 2.36 ^a^

The data presented with various superscripts such as a, b, and c reflect significant differences (*p* < 0.05) (*n* = 4).

**Table 4:** Growth performance during a planting period of 90 days.

90 Days	Plant Height (cm)	Stem Diameter (mm)	Flower Count	Fruit Count
T0	89.40 ± 6.92 ^c^	7.85 ± 0.80 ^c^	0.66 ± 0.98	1.41 ± 1.37 ^b^
T1	116.71 ± 11.87 ^b^	9.56 ± 0.89 ^b^	1.25 ± 1.31	2.00 ± 1.27 ^ab^
T2	121.08 ± 15.65 ^b^	9.30 ± 1.16 ^b^	1.00 ± 1.21	2.91 ± 1.92 ^a^
T3	149.73 ± 15.09 ^a^	12.15 ± 0.95 ^a^	1.41 ± 1.16	2.91 ± 1.97 ^a^

The data presented with various superscripts such as a, b, and c reflect significant differences (*p* < 0.05) (*n* = 4).

**Table 5:** Growth performance and nutritional composition of mirror carp (*Cyprinus carpio var. specularis*) at 30 days WGR (%).

30 Days	T0	T1	T2	T3
Body length (cm)	10.92 ± 0.42	11.03 ± 0.15	11.07 ± 0.71	10.88 ± 0.45
Body weight (g)	28.91 ± 4.05	29.78 ± 1.29	28.88 ± 1.16	29.96 ± 1.84
WGR (%)	33.48 ± 0.26	37.50 ± 5.98	33.36 ± 5.39	38.35 ± 8.53
SGR (%/day)	1.28 ± 0.30	1.38 ± 0.14	1.28 ± 0.13	1.40 ± 0.20
PER (%)	17.69 ± 0.63	19.81 ± 3.16	17.62 ± 2.84	20.26 ± 4.51
CF (g/cm^3^)	2.65 ± 0.10	2.70 ± 0.13	2.62 ± 0.06	2.75 ± 0.13
FCR	1.79 ± 0.19	1.62 ± 0.25	1.82 ± 0.29	1.61 ± 0.34
Moisture content	77.79 ± 3.1	77.99 ± 1.01	75.16 ± 3.28	76.5 ± 1.89
Ash content	2.06 ± 0.2	2.18 ± 0.22	2.22 ± 0.32	2.26 ± 0.6
Crude protein	15.04 ± 0.21	15.59 ± 0.44	15.30 ± 0.14	15.63 ± 0.57
Crude fat	2.70 ± 0.68	3.22 ± 0.61	3.05 ± 0.93	3.16 ± 1.45

There is no significant difference in data between each group (*p* < 0.05) (*n* = 3).

**Table 6:** Growth performance and nutritional composition of mirror carp (*Cyprinus carpio var. specularis*) at 60 days WGR (%).

60 Days	T0	T1	T2	T3
Body length (cm)	13.65 ± 0.91	13.48 ± 0.70	13.66 ± 0.18	13.93 ± 0.20
Body weight (g)	40.10 ± 4.62	37.52 ± 1.28	38.93 ± 1.18	39.95 ± 1.02
WGR (%)	38.70 ± 16. 00	25.98 ± 4.30	27.45 ± 3.86	33.40 ± 3.55
SGR (%/day)	0.89 ± 0.06	1.18 ± 0.17	1.18 ± 0.08	0.92 ± 0.08
PER (%)	20.44 ± 8.45	13.72 ± 2.27	14.49 ± 2.04	16.84 ± 1.79
CF (g/cm^3^)	2.93 ± 0.28	2.78 ± 0.08	2.85 ± 0.06	2.86 ± 0.10
FCR	1.80 ± 0.92	2.35 ± 0.38	2.21 ± 0.33	1.89 ± 0.20
Moisture content	77.80 ± 1.2	77.85 ± 0.61	78.23 ± 0.91	77.61 ± 0.58
Ash content	1.96 ± 0.31	2.03 ± 0.67	2.28 ± 0.54	2.14 ± 0.35
Crude protein	15.31 ± 0.3	15.07 ± 0.72	15.51 ± 0.16	16.16 ± 0.89
Crude fat	3.17 ± 0.66	2.33 ± 0.17	3.20 ± 0.38	3.22 ± 0.62

There is no significant difference in data between each group (*p* < 0.05) (*n* = 3).

**Table 7:** Growth performance and nutritional composition of mirror carp (*Cyprinus carpio var. specularis*) at 90 days WGR (%).

90 Days	T0	T1	T2	T3
Body length (cm)	14.78 ± 0.30	14.84 ± 0.29	14.64 ± 0.39	14.56 ± 0.20
Body weight (g)	47.83 ± 2.51 ^a^	47.08 ± 0.83 ^a^	45.93 ± 1.03 ^a^	41.92 ± 1.41 ^b^
WGR (%)	19.27 ± 6.28 ^a^	25.48 ± 2.27 ^a^	17.96 ± 2.64 ^a^	4.93 ± 3.53 ^b^
SGR (%/day)	0.65 ± 0.17 ^a^	0.82 ± 0.05 ^a^	0.62 ± 0.07 ^a^	0.23 ± 0.10 ^b^
PER (%)	10.18 ± 3.31 ^a^	13.46 ± 1.20 ^a^	9.49 ± 1.39 ^a^	2.65 ± 1.86 ^b^
CF (g/cm^3^)	3.23 ± 0.11 ^a^	13.46 ± 1.20 ^a^	3.16 ± 0.02 ^a^	2.87 ± 0.07 ^b^
FCR	3.40 ± 1.36 ^b^	2.36 ± 0.20 ^b^	3.88 ± 0.49 ^b^	11.94 ± 1.74 ^a^
Moisture content	77.79 ± 3.1	77.99 ± 1.01	75.16 ± 3.28	76.5 ± 1.89
Ash content	2.06 ± 0.2	2.18 ± 0.22	2.22 ± 0.32	2.26 ± 0.6
Crude protein	16.84 ± 1.40	17.17 ± 0.71	15.23 ± 0.9	15.14 ± 0.39
Crude fat	3.99 ± 0.28 ^ab^	4.36 ± 1.03 ^a^	4.15 ± 0.86 ^b^	3.99 ± 0.42 ^b^

The data presented with various superscripts such as a and b reflect significant differences (*p* < 0.05) (*n* = 3).

## Data Availability

The data presented in this study are available in this article.
